# Microwave Annealing for NiSiGe Schottky Junction on SiGe P-Channel

**DOI:** 10.3390/ma8115403

**Published:** 2015-11-10

**Authors:** Yu-Hsien Lin, Yi-He Tsai, Chung-Chun Hsu, Guang-Li Luo, Yao-Jen Lee, Chao-Hsin Chien

**Affiliations:** 1 Department of Electronic Engineering, National United University, Miaoli 36003, Taiwan; a246792001@yahoo.com.tw; 2 Department of Electronics Engineering and Institute of Electronics, National Chiao-Tung University, Hsinchu 30010, Taiwan; arthur00403@hotmail.com (C.-C.H.); chchien@faculty.nctu.edu.tw (C.-H.C.); 3 National Nano Device Laboratories, Hsinchu 30010, Taiwan; glluo@narlabs.org.tw (G.-L.L.); yjlee@ndl.narl.org.tw (Y.-J.L.)

**Keywords:** germanium, microwave annealing, NiSiGe, Schottky junction

## Abstract

In this paper, we demonstrated the shallow NiSiGe Schottky junction on the SiGe P-channel by using low-temperature microwave annealing. The NiSiGe/n-Si Schottky junction was formed for the Si-capped/SiGe multi-layer structure on an n-Si substrate (Si/Si_0.57_Ge_0.43_/Si) through microwave annealing (MWA) ranging from 200 to 470 °C for 150 s in N_2_ ambient. MWA has the advantage of being diffusion-less during activation, having a low-temperature process, have a lower junction leakage current, and having low sheet resistance (Rs) and contact resistivity. In our study, a 20 nm NiSiGe Schottky junction was formed by TEM and XRD analysis at MWA 390 °C. The NiSiGe/n-Si Schottky junction exhibits the highest forward/reverse current (I_ON_/I_OFF_) ratio of ~3 × 10^5^. The low temperature MWA is a very promising thermal process technology for NiSiGe Schottky junction manufacturing.

## 1. Introduction

As the devices are being continuously scaled down for logic circuits, higher mobility channel materials such as Ge or SiGe have been considered to boost the driving current [1,2,3,4,5]. However, most high mobility materials have a significantly smaller bandgap as compared to Si, which will result in a higher band-to-band tunneling leakage. Therefore, S/D (Source/Drain) and channel engineering must play leading roles for boosting device performance. First, the parasitic series resistance should be reduced. The low dopant solid solubility in Ge results in the large S/D series resistance. A large S/D series resistance can be restrained by introducing metal germanide S/D. Second, the shallow junction is needed. The interface between the metal and the semiconductor is abrupt and can be easily governed by the reactant metal thickness and the process thermal budget, which indicates a high potential for scalability [6]. Third, simpler device fabrication could be achieved for the Schottky device without ion implantations and the high temperature annealing for dopant activation [7,8].

NiSiGe is the most promising candidate due to its low resistivity for the junction contact [9,10,11,12]. For reducing the thickness of the NiSiGe layer of the Schottky junction, the process temperature needs to be reduced. However, a lower process temperature results in a higher NiSiGe resistance due to the small crystallite size of the NiSiGe layer. These are the major challenges of scaling Ge CMOS (Complementary Metal-Oxide-Semiconductor) into nanoscale devices. Therefore, in order to avoid the dopant diffusion effect, which is dominant at high annealing temperatures, low-temperature annealing with microwave excitation appears to offer a promising microwave annealing (MWA) process that may be an alternative to other rapid thermal processing methods in silicon processing [13,14,15]. Microwaves could repair the damage in the Schottky junction formation and provide the lower leakage current for the Schottky junction device.

In this paper, we propose a NiSiGe/n-Si Schottky junction formed by microwave annealing in the Si-capped SiGe Schottky junction devices (Si/Si_x_Ge_1-x_/Si, x = 0~1). The shallow junction with a 20 nm depth has been fabricated with a high effective barrier height (${\text{ϕ}}_{\text{B}}^{\mathrm{eff}}$) and low leakage current in the devices.

## 2. Experimental Section

**Figure 1:**
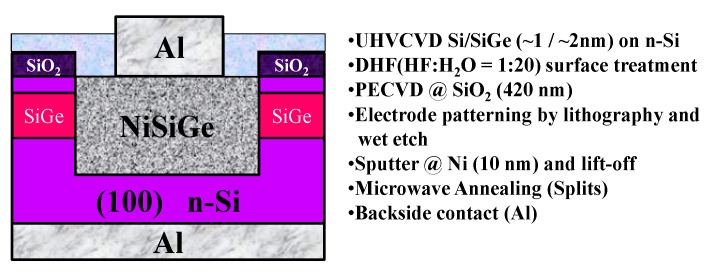
Schematic diagram and process flow of fabricating the NiSiGe/n-Si Schottky junction structure.

Figure 1 shows the schematic diagram and process flow of fabricating the NiSiGe/n-Si Schottky junction structure. The Schottky junction devices were fabricated on a four-inch silicon wafer. The multi-layer structure of Si/Si_0.57_Ge_0.43_ (1 nm/2 nm/n-Si) was grown by an ultra-high vacuum chemical vapor deposition system (UHVCVD, CANON ANELVA Corporation (Kanagawa, Japan)) on an n-type Si substrate. The channel was composed of a 1-nm-thick Si cap and a 2-nm-thick SiGe layer with biaxial compressive strain and was grown at 420–500 °C and 550 °C, respectively. The isolation film of 420 nm SiO_2_ was deposited on the multi-layer architecture after series surface cleaning. Then, the definition of the junction active area was accomplished with lithography and wet-etching. Because of the bulk annealing characteristics of the microwave, the technique was utilized to form NiSiGe as a low-leakage Schottky junction ranging from 200 to 470 °C for 150 s in N_2_ ambient. The un-reacted Ni film was removed, followed by Al deposition as the back contact.

## 3. Results and Discussion

**Figure 2:**
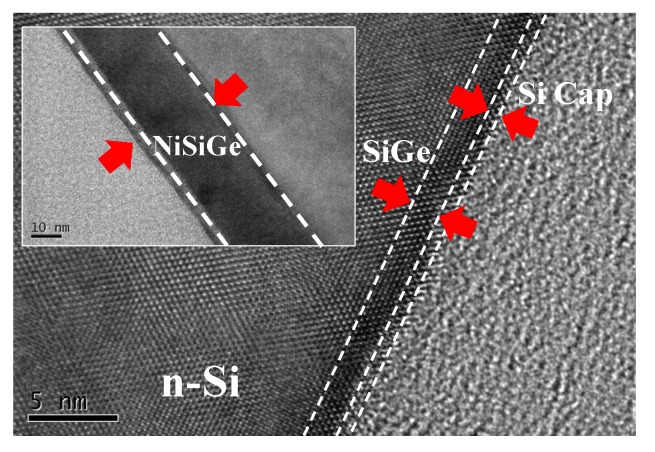
The cross-sectional TEM images show the polycrystalline structure in the Si/SiGe/n-Si lattice interface image. The inset figure shows the NiSiGe Schottky junction through MWA at 390 °C.

Figure 2 shows the high resolution TEM images of an approximately 1 nm/2 nm Si/SiGe film for the multi-layer Si/Si_0.57_Ge_0.43_/n-Si structure before MWA. The SiGe/Si lattice interface image shows the good polycrystalline structure. The inset figure shows the NiSiGe film after MWA at 390 °C. A 20-nm relative uniformity of the NiSiGe film and a distinct interface between the NiSiGe and Si could be observed.

**Figure 3:**
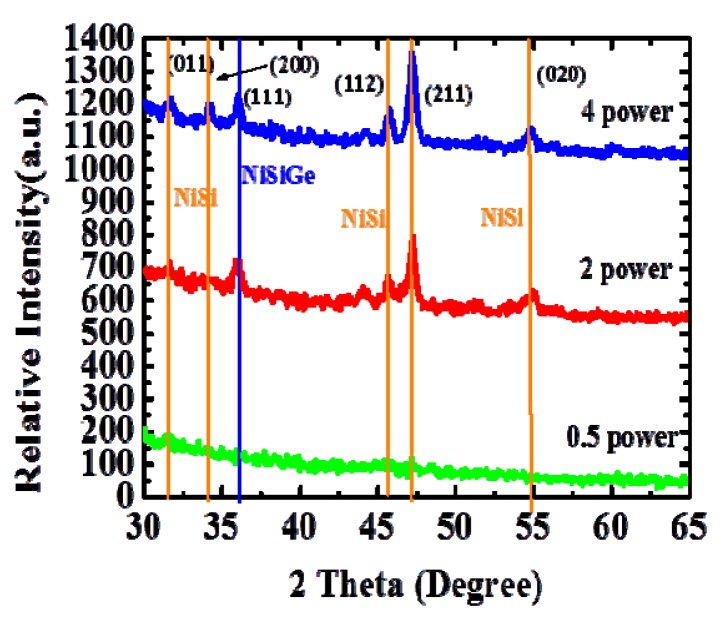
The GIXRD spectra for MWA with different power splits, confirming the NiSiGe formation.

The result of the grazing incidence of X-ray diffraction (GIXRD) analysis was shown in Figure 3. From this figure, the descriptions of power 0.5, power 2, power 4 are 300 W, 1200 W, 2400 W, respectively. Moreover, the temperature measurements of the above power splits are 200 °C, 390 °C, and 470 °C, respectively. Many peaks are shown, which correspond to the crystalline nickel monogermanide; implementing 390 °C for 150 s MWA is sufficient to form polycrystalline NiGe and NiSiGe. The peaks corresponding to (011), (200), (112), (211), and (020) NiSi and (111) NiSiGe were clearly identified [16]. Performing the MWA at 390 °C for 150 s was sufficient to form the NiSi and NiSiGe phase for forming the Schottky junction.

**Figure 4:**
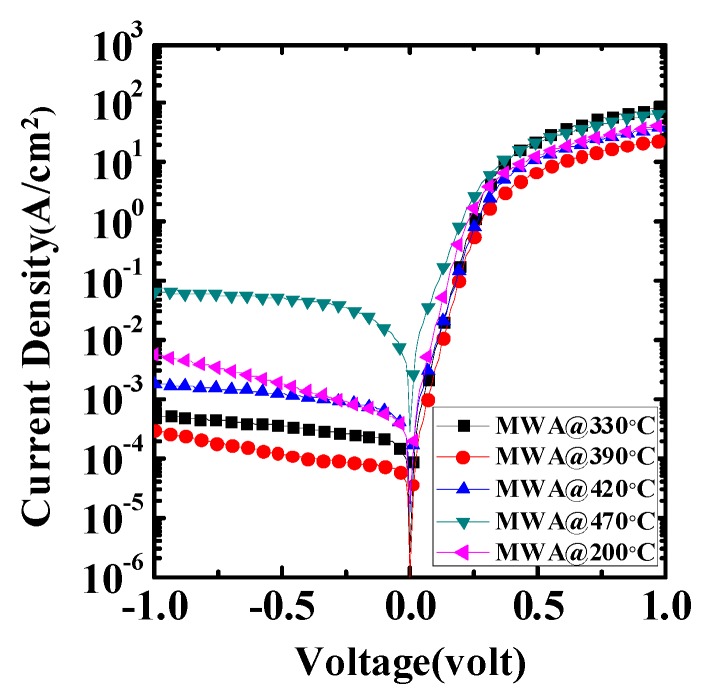
The *I*–*V* characteristics of the NiSiGe/n-Si Schottky junction annealed at different MWA temperatures.

Figure 4 shows the *I*–*V* characteristics of the fabricated NiSiGe/n-Si Schottky junction; the NiSiGe formation during MWA conditions was at 200 °C, 330 °C, 390 °C, 420 °C, and 470 °C for 150 s, respectively. After forming NiSiGe with MWA annealing, the forward currents and reverse leakage currents of all NiSiGe/n-Si contacts gradually decreased from 200 °C to 390 °C. However, increasing the annealing temperature from 200 °C to 470 °C, the forward currents and reverse leakage currents are degraded accordingly. The NiSiGe/n-Si Schottky junction exhibits the highest forward/reverse current ratio of ~2.5 × 10^5^ at MWA 390 °C. This result also indicates that the series resistance can be significantly reduced after the SiNiGe formation at the condition of MWA 390 °C. Note that if using relatively high temperature MWA annealing at >470 °C, the crystallization became more significant and which was shown by the increased intensity of the GIXRD peak in Figure 3. This issue will potentially degrade the uniformity, cause more defects induce at the interface, and affect the leakage current of the junction increase.

**Figure 5:**
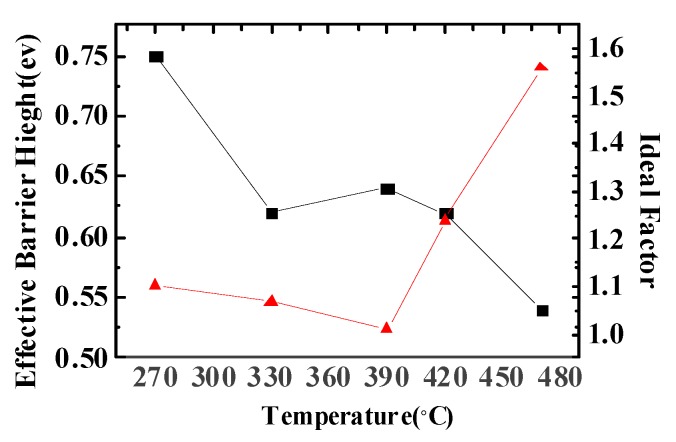
SBH and ideality factor of NiSiGe/n-Si Schottky contact with different MWA temperatures.

Figure 5 shows the effective electron SBH (Schottky barrier height) and ideality factor of NiSiGe/n-Si Schottky junctions with different MWA temperatures. For a typical or moderate doped semiconductor, the *I*–*V* characteristics of the Schottky diode could be described by:

(1) $$I=I_{S}\exp\left(\frac{qV_{a}}{nK_{B}T}-1\right)$$

with

(2) $$I_{S}=AA^{*}T^{2}\exp\left(\frac{q\phi_{B}^{eff}}{K_{B}T}\right)$$

where *I_s_* is the saturation current, *A* is the diode area, *V_a_* is the applied voltage, A^*^ is the effective Richardson constant [17,18], ${\text{ϕ}}_{\text{B}}^{\mathrm{eff}}$ is the SBH, and *n* is the ideality factor. The ideality factor *n* and SBH ${\text{ϕ}}_{\text{B}}^{\mathrm{eff}}$ can be derived as:

(3) $$n=\left(\frac{q}{K_{B}T}\right)\left(\frac{\partial V}{\partial [\ln I]}\right)$$

and

(4) $$\phi_{B}^{eff}=\frac{K_{B}T}{q}\ln\left(\frac{A^{*}T^{2}}{J_{S}}\right)$$

By using Equation (3), we extract the ideal factor *n* and SBH ${\text{ϕ}}_{\text{B}}^{\mathrm{eff}}$ from each MWA sample of a different temperature in the I–V characteristics of Figure 4. From Figure 5, we can observe that the SBH and the ideality factor of the MWA 390 °C condition are 0.63 eV and 1.01, and it shows good electrical characteristics of Schottky junctions.

## 4. Conclusions

This paper realized the shallow NiSiGe Schottky junction on the SiGe P-channel by using low-temperature microwave annealing. The formation of junction defects could be suppressed and prevent the agglomeration due to the lower forming temperature. The microwave-annealed NiSiGe Schottky junction exhibited a superior I_ON_/I_OFF_ ratio of about 3 × 10^5^ formed at 390 °C as well as more stable off-current characteristics with an SBH of 0.63 eV and an ideality factor of 1.01. We believe our microwave annealing NiSiGe Schottky junction is promising for high performance logic circuits and will enable SiGe channel devices to be integrated on the Si substrate for the future applications.
